# Pharmacogenetics of thiopurines for inflammatory bowel disease in East Asia: prospects for clinical application of NUDT15 genotyping

**DOI:** 10.1007/s00535-017-1416-0

**Published:** 2017-11-30

**Authors:** Yoichi Kakuta, Yoshitaka Kinouchi, Tooru Shimosegawa

**Affiliations:** 1 Division of Gastroenterology, Tohoku University Graduate School of Medicine, 1-1 Seiryo, Aoba, Sendai, 980-8574 Japan; 2 Institute for Excellent in Higher Education, Tohoku University, Sendai, Japan

**Keywords:** Azathiopurine, 6-Mercaptopurine, NUDT15, Pharmacogenetics, Inflammatory bowel disease

## Abstract

The thiopurine drugs 6-mercaptopurine (6-MP) and azathiopurine (AZA) are widely used to treat inflammatory bowel disease. However, the incidence of adverse reactions is high, particularly in Asia, and the mechanisms of toxicity in Asian populations remain unclear. Thiopurine *S*-methyltransferase (TPMT) is a well-known enzyme that inactivates AZA or 6-MP through methylation and is one of the few pharmacogenetic predictors used in clinical settings in Western countries. Individuals carrying TPMT-deficient genetic variants require reduced drug doses, but this treatment modification is are not applicable to East Asian populations. Several genes code thiopurine-metabolizing enzymes, including TPMT, multidrug-resistance protein 4, and inosine triphosphatase. These genes have been studied as candidate pharmacogenetic markers; however, it remains unclear why Asian populations seem to be more intolerant than other ethnic groups to a full dose of thiopurines. A genome-wide association approach to identify Asian-specific pharmacogenetic markers in Korean patients with Crohn’s disease revealed that a non-synonymous single nucelotide polymorphism in nucleoside diphosphate-linked moiety *X*-type motif 15 (NUDT15) which causes p.Arg139Cys was strongly associated with thiopurine-induced early leukopenia. Six common haplotypes of NUDT15 were reported, and five variants showed medium-to-low enzyme activities, compared with the wild haplotype. NUDT15 hydrolyzes the thiopurine active metabolites 6-thio-GTP and 6-thio-dGTP; variants of NUDT15 had lower enzyme activities, causing higher levels of thiopurine active metabolites, resulting in thiopurine-induced leukopenia. In clinical application, NUDT15 genotyping is a good candidate for predicting thiopurine toxicity in East Asian populations. However, the association of NUDT15 diplotypes with thiopurine toxicity remains unclear. Further analyses with large cohorts to confirm the clinical effects of each haplotype are planned.

## Introduction

Inflammatory bowel diseases (IBDs), represented by ulcerative colitis and Crohn’s disease, are chronic inflammatory intestinal conditions of unknown etiology. The thiopurine drug, 6-mercaptopurine (6-MP), and its pro-drug, azathiopurine (AZA), are widely used to treat IBD, and thiopurines are well established as key drugs for sparing steroid therapy and maintaining remission of IBD [1–6]. Additionally, the combined treatment of anti-tumor necrosis factor (TNF) agents with thiopurines has been shown to reduce the risk of anti-drug antibody formation that may diminish response to anti-TNF agents [7–9].

Despite the efficacy of thiopurines, the incidence of adverse reactions is high, particularly in East Asian populations, including Koreans [10], Chinese [11], and Japanese [3, 12]. There are definite racial differences in adverse reaction profiles [13, 14]. For example, the incidence of leukopenia is higher in Asian populations than in Caucasian populations, and hair loss is not uncommon in Japanese patients but very rare in Caucasians, although the standard dose of thiopurines in Japan (AZA 1–2 mg/kg/day) is less than one-half of that used in Europe (AZA 2–2.5 mg/kg/day) [15]. Some of these adverse reactions are known to be caused by individual differences in thiopurine metabolism, which is affected by the genetic polymorphism of the enzymes [16, 17]. Several pharmacogenetic studies, not only for IBD but also for leukemia and organ transplantation, have been reported [18, 19], and a few of the pharmacogenetic predictors thus identified have been applied in clinical settings [20–22].

Given this background, in this review we focus on recent advances in pharmacogenetic research of thiopurines and the prospects for clinical application of pharmacogenetic markers, paying attention to racial differences.

## Thiopurine metabolic pathway

**Fig. 1:**
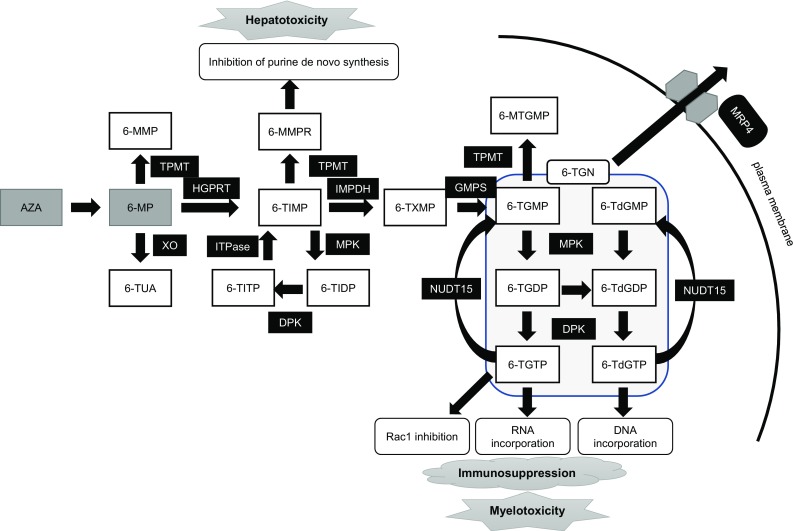
Thiopurine metabolism and transportation. Drugs are shown in gray boxes:* AZA* azathioprine,* 6-MP* 6-mercaptopurine. Metabolites are shown in white boxes:* 6-MMP* 6-Methylmercaptopurine,* 8-OHMP* 8-hydroxy-6-mercaptopurine,* 6-TUA* 6-thiouric acid,* 6-MMPR* 6-methylmercaptopurine ribonucleotides,* 6-TIMP* 6-thioinosine monophosphate,* 6-TIDP* 6-thioinosine diphosphate,* 6-TITP* 6-thioinosine triphosphate,* 6-TXMP* 6-thioxanthosine monophosphate,* 6-TGMP* 6-thioguanine monophosphate,* 6-TGDP* 6-thioguanine diphosphate,* 6-TGTP* 6-thioguanine triphosphate,* 6-TdGMP* 6-thio-deoxyguanine monophosphate,* 6-TdGDP* 6-thio-deoxyguanine diphosphate,* 6-TdGTP* 6-thio-deoxyguanine triphosphate,* 6-MTGMP* 6-methylthioguanine monophosphate,* 6-TGN* 6-thioguaninenucleotides. Enzymes or transporters are shown in black boxes:* XO* xanthine oxidase,* TPMT* thiopurine* S*-methyl transferase,* HGPRT* hypoxanthine phosphoribosyl transferase,* IMPDH* inosine monophosphate dehydrogenase,* GMPS* guanosine monophosphate synthetase,* MPK* monophosphate kinase,* DPK* diphosphate kinase,* ITPase* inosine triphosphate pyrophosphatase,* MRP4* multidrug resistance-associated protein 4

The metabolic pathways of AZA and 6-MP are well described. These substances are metabolized into their active metabolites through a series of steps [23–26], and 6-thioguanine nucleotides (6-TGNs) consist of 6-T(d)GMP, 6-T(d)GDP, and 6-T(d)GTP. 6-T(d)GTPs are incorporated into DNA (6-TdGTP) and RNA (6-TGTP), causing inhibition of nucleotide and protein synthesis [27, 28] and resulting in immunosuppression. 6-TGTPs also block the Vav-Rac1 pathway in T-cells, inhibiting T-cell–antigen-presenting cell conjugation and the subsequent immune responses [29]. As shown in Fig. 1, there are several thiopurine-metabolizing enzymes, and their activities are partly defined by their genetic polymorphisms. Lower activity of the enzymes that inactivate thiopurines, such as thiopurine *S*-methyltransferase (TPMT), leads to higher production of 6-TGNs or other metabolites [31, 32], causing dose-dependent adverse reactions typified by leukopenia [16, 18, 19].

## Thiopurine *S*-methyltransferase

Thiopurine *S*-methyltransferase is a well-known enzyme that inactivates AZA or 6-MP by methylation; it is one of the few pharmacogenetic predictors used in clinical settings. TPMT deficiency causes increasing 6-TGN levels related to leukopenia [16, 32] and increasing 6-methylmercaptopurine (6-MMP) levels and 6-MMPR levels related to hepatotoxicity [33–36]. There are two ways of testing for TMPT deficiency: enzyme activity or genotype [30]. The first report of inter-individual variations in TPMT enzyme activity identified three levels of activity, namely, high, intermediate, and deficient; in the Caucasian populations tested, approximately 89% had high enzyme activity and 11% had intermediate activity, whereas only 0.3% were deficient [30]. This classification scheme is widely known, but TPMT enzyme activities have since been recognized to be more variable [37]. Genetic polymorphisms cause TPMT deficiency, and more than 40 different variant* TPMT* alleles (TPMT*2–*41) have been reported up to May 2017 [38, 39]. Most of these variants are associated with decreased TPMT activity, relative to the wild allele (TPMT*1) [40]. Major TPMT mutant alleles for decreased TPMT activity are TPMT*2, TPMT*3A, and TPMT*3C in most populations, with other variants being rare.

The Clinical Pharmacogenetics Implementation Consortium published dosing recommendations for thiopurines based on TPMT genotype [22]. According to these guideline, patients who are heterozygous for the *1 allele and demonstrate reduced activity of the of *2/*3A/*3C/*4 alleles (intermediate methylators) should receive 30–70% of the full dose (AZA 1–1.5 mg/kg/day). Those who are homozygous for the alleles showing reduced activity (deficient methylators) should receive 10% of the full dose at a reduced frequency (every other day administration). TMPT deficiencies that are derived from genetic polymorphisms are robust markers of thiopurine-induced leukopenia. Despite the success of this pharmacogenetic test, a number of major issues remain. One is that the TPMT activity is not defined by a common genotype only [41]. As already mentioned, there are many genotypes of TPMT, and it is difficult to determine a patient’s exact genotype using a commercial test. Additionally, there are novel and/or rare variants in coding regions that affect TPMT activity [39, 42, 43], as well as other genetic variants located in non-coding regions that affect the expression and/or activity levels of the* TPMT* gene [44, 45]. Furthermore, several co-factors controlling TPMT activities are affected by genetic variations of related genes. Inhibition of the folate cycle affects TPMT activities [46, 47], and genetic variants of methylenetetrahydrofolate reductase (MTHFR), which is associated with folate metabolism, are also associated with TPMT activity [48, 49]. Thus, measuring TPMT activity is a more accurate strategy for predicting the appropriate dose of thiopurines than is TPMT genotyping.

Another issue is ethnicity. The frequencies of these genetic polymorphisms vary in different ethnic groups. A recent extensive whole-genome resequencing of 3554 Japanese individuals found that alleles *3A and *3B were not present (not observed) and that only the presence of *3C could be confirmed (0.96%) [50, 51]. Uchiyama et al. reported that TPMT mutant alleles were not detected in 16 intolerant Japanese patients and that TPMT genotypes were not associated with thiopurine-induced leukopenia in a Japanese cohort [52]. Even though there are some very rare variants, such as *26 and *30 in Chinese and Japanese populations [40, 53, 54], TPMT deficiency cannot explain the higher incidence of adverse reactions in East Asian patients.

## Multidrug-resistance protein 4 and inosine triphosphatase

In light of results indicating the absence or low frequency of the* TPMT* gene in Japanese populations [12, 52, 55], there must be other genetic variations that could explain the frequent thiopurine-induced leukopenia in East Asian populations. Multidrug-resistance protein 4 (*MRP4*; also known as ABCC4) is a member of a family of multi-specific drug transporters and a candidate gene associated with thiopurine metabolism [56]. Mrp4-deficient mice were found to experience thiopurine-induced hematopoietic toxicity caused by the accumulation of 6-TGNs in their myelopoietic cells [57]. MRP is an ATP-dependent efflux pump, therefore Mrp4 may protect thiopurine toxicity by exporting thiopurine metabolites.* MRP4*-G2269A (rs3765534) causes* MRP4* deficiency [57]. 6-TGN levels were found to be significantly higher in patients with* MRP4*-G2269A, resulting in a significant association with thiopurine-induced leukopenia in Japanese patients with IBDs [58]. However, the* MRP4* variant alone could not explain the frequently observed thiopurine toxicity in Asian populations. Interestingly, the* MRP4* variant was reported to show gene–gene interactions with inosine triphosphatase (ITPA) and nucleoside diphosphate-linked moiety *X*-type motif 15 (NUDT15) variants [58–60]. Variants of the* ITPA* gene are also candidate predicting markers for adverse events. ITPA is widely expressed in leukocytes and erythrocytes [61, 62], and it catalyzes the hydrolysis of ITPA to prevent the accumulation of 6-thioinosine triphosphate (6-TITP) [61]. 6-TITP would be incorporated into DNA and RNA and compete with nucleotides similar to 6-T(d)GTP of the 6-TGNs. The single nucleotide polymorphisms (SNPs) 94C > A and IVS2 + 21A > C, which are associated with ITPA deficiency, have also been found to be associated with thiopurine intolerance, such as leukopenia, flu-like symptoms, and pancreatitis [63, 64]. These toxicities could be caused by high 6-TITP levels. In Japanese patients with IBD, Uchiyama et al. reported that the ITPA 94C > A polymorphism was frequent in those patients with thiopurine-induced adverse effects, including leukopenia [52]. Alternatively, ITPA deficiency could reduce the levels of 6-TGNs by reducing the conversion from 6-TITP to 6-thioinosine monophosphate (6-TIMP), resulting in re-entry into the 6-MP metabolism pathway. Ban et al. reported that Japanese patients carrying the* ITPA* 94C > A polymorphism showed significantly lower 6-TGN levels, without leukopenia [58]. Patients carrying both of these* MRP4* and* ITPA* variants did not show leukopenia, and it is possible that the leukopenia associated with *MRP4* variants was masked by the opposing effects of ITPA deficiency. The association of ITPA variants with thiopurine toxicity is controversial [65–67], and the impact of ITPA variants does not appear to favor clinical application.

## Nucleoside diphosphate-linked moiety *X*-type motif 15

In addition to TPMT, other genes, including* MRP4* and ITPA, have been studied as candidates in Asian populations [10, 12, 52, 55, 68, 69]. However, these genes cannot explain the fact that Asian populations seem to be more intolerant to full doses of thiopurines than Caucasian ones. Therefore, population-specific genetic variants associated with thiopurine intolerance appear to exist. A breakthrough was achieved through a genome-wide association study of Korean patients with Crohn's disease. Yang et al. reported that a non-synonymous SNP in NUDT15 (also known as MTH2) that causes p.Arg139Cys (R139C) was very strongly associated with thiopurine-induced early leukopenia in Koreans [odds ratio (OR) 35.6; *P* = 4.88 × 10^−94^]; these authors also confirmed this association in patients with IBD who were of European ancestry (OR 9.50; *P* = 4.64 × 10^−4^), although it was very rare (minor allele frequency < 0.004) [70]. NUDT15 p.Arg139Cys had a high sensitivity (89.4%) and specificity (93.2%) for early leukopenia. The association was also confirmed in populations in Japan, China, India, Thai, Singapore, Guatemala, and Uruguay [71–81]. Furthermore, we have reported that almost all of the patients homozygous for p.Arg139Cys had severe early leukopenia and severe alopecia [72]. Alopecia is known to be a major severe complication in Asian populations, but it is very rare in individuals of European ancestry. Patients who were homozygous for NUDT15 p.Arg139Cys had nearly perfect sensitivity and specificity (≈ 100%) for severe alopecia, resembling “Mendelian” drug intolerance [72, 73, 82]. Taking into account the results from previous studies, thiopurines would appear to be contraindicated in patients with IBD who are homozygous for p.Arg139Cys (genotype TT). The incidence of early leukopenia (< 8 weeks) in patients with IBD who are heterozygous for p.Arg139Cys (genotype CT) was found to be significantly higher than that of patients who are homozygous for the wild-allele (genotype CC) (17.4 vs. 0.93%); however, there were no significant differences in the continuous rates of thiopurines between genotype CT and CC upon dose manipulation [72]. Among Japanese patients with IBD, the average maintenance dose of patients with the CT genotype was almost one-half of that of the patients with the CC genotype (0.574 ± 0.316 vs. 1.03 ± 0.425 mg/kg/day, respectively) [72]. Therefore, testing the genotype of NUDT15 p.Arg139Cys could be useful not only for detecting patients with contraindications for thiopurines (genotype TT), but also to optimize the initial dose of thiopurines.

## What does NUDT15 do?

Most of previously reported genes are associated with thiopurine-induced leukocytopenia with elevated 6-TGN levels. However, Asada et al. reported that there were no differences in 6-TGN levels in patients with each genotype of NUDT15 p.Arg139Cys [73]. Therefore, NUDT15 p.Arg139Cys-related thiopurine-induced leukocytopenia is likely mediated by a 6-TGN-independent mechanism [73, 74, 83]. At the time when the first genome-wide association study was reported, NUDT15 was considered to affect 8-oxo-dGTPase based on the results of only one 2003 study [84]. In 2015, Carter et al. reported that NUDT15 had no effect on the incorporation of 8-oxo-dGTP into DNA and that it hydrolyzes 6-thio-dGTP, 6-thio-GTP, and dGTP [85]. Interestingly, NUDT15 hydrolyzes 6-thio-dGTP and 6-thio-GTP more efficiently than it does GTP and dGTP. Moriyama et al. reported that NUDT15 converted the thiopurine active metabolites 6-thio-GTP and 6-thio-dGTP into 6-thio-GMP and 6-thio-dGMP and that the variants of NUDT15 had lower enzyme activity [80]. This caused higher thiopurine active metabolite levels, resulting in thiopurine-induced leukopenia [80]. Therefore, the DNA-incorporated thioguanine (DNA-TG) metabolite was preferable to 6-TGN for adjusting thiopurine doses according to NUDT15 genotypes [86]. Valerie et al. reported that the enzyme efficiencies of the p.Arg139Cys variants were similar to that of the wild-type, but that p.Arg139Cys caused structural abnormalities and the mutant rapidly degraded in cells [87].

## Additional variants of NUDT15 and their enzymatic activities

**Fig. 2:**
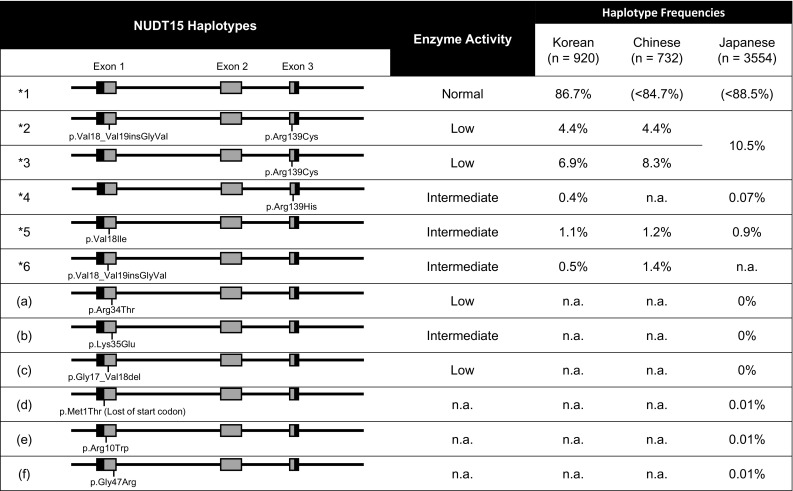
Nucleoside diphosphate-linked moiety *X*-type motif 15 (NUDT15) haplotype structures and frequencies in East Asian populations. Common haplotypes *1 to *6 have been defined previously [81]. Rare variants (a), (b), (c) are from Moriyama et al. [92], and rare variants (d), (e), and (f) and estimated haplotype frequencies in Japanese populations were from the 3.5KJPN data by the Tohoku Medical Megabank Project (https://ijgvd.megabank.tohoku.ac.jp/) [51, 52, 91].* n.a.* data not available

Moriyama et al. studied major haplotypes of NUDT15 and their enzyme activities by analyzing 270 children with acute lymphoblastic leukemia (ALL) in Guatemala, Singapore, and Japan [80]. These authors identified four coding variants in exons 1 and 3, including the previously reported p.Arg139Cys in exon 3, and defined six haplotype (*1 to *6) combinations of these four variants (Fig. 2). Three variants, Arg139His, Val18Ile, and p.Val18_Val19insGlyVal, reduce NUDT15 enzyme activity at similar levels (almost 75%); no enzyme activity was observed in the p.Arg139Cys variant. Haplotype frequencies in the Korean cohort [88] and in Chinese patients with IBD [89], and estimated frequencies of the Japanese cohort study for the Tohoku Medical Megabank Project [50, 51, 90] are summarized in Fig. 2. Individual haplotype and insertion/deletion data of the Japanese cohort were not available; therefore, the frequencies are based on single-nucleotide variant frequencies. Distributions of the haplotype frequencies were similar in these three populations, and the two variants in exon 1 are not rare in East Asian populations. The diplotype (combination of haplotypes that exist in each individual) is more important in clinical settings. Moriyama et al. classified patients into three diplotypic groups according to expected enzyme activities: normal (*1/*1), intermediate (*1/*2, *1/*3, *1/*4, and *1/*5), and low activity (*2/*3, *3/*3, and *3/*5) [80]. In their study, they showed differences in the tolerated 6-MP doses for each population. However, there were limited numbers of subjects in each population, and haplotype *6 and some of the estimated diplotypes were not observed. Moreover, the treatment goals and dosages of 6-MP for ALL treatments in children are very different from those required in patients with IBD; therefore it is difficult to apply this classification directly to patients with IBD. Chao et al. recently reported associations between leukopenia and the NUDT15 diplotype in Chinese patients with IBD [89]. In this study, patients with *1/*6 (*n* = 20) or *2/*6 diplotype (*n* = 1) were observed. The frequencies of leukopenia in these patients were 35.0 and 100%, respectively, which is higher than the 15.1% in patients with the *1/*1 diplotype. The authors reported that the leukopenia frequencies appeared to be clearly related to NUDT15 enzyme activity based on diplotypes (Table 1).

**Table 1:** Nucleoside diphosphate-linked moiety *X*-type motif 15 diplotypes and leukopenia frequencies

NUDT15 enzyme activity [81]	Normal	Medium					Low					
Diplotype	*1*1	*1*4	*1*5	*1*6	*1*2	*1*3	*2*6	*2*5	*3*5	*2*2	*2*3	*3*3
Diplotype frequencies												
Chinese [90] (%)	< 71.6	na	1.78	2.73	7.8	13.9	0.14	0	0.55	0	0.82	0.68
Korean (estimated)^a^ (%)	75.2	0.69	1.9	0.87	7.6	12.0	0.044	0.10	0.15	0.19	0.61	0.48
Japanese (estimated)^b^ (%)	< 78.3	0.12	1.6	n.a.	18.6		n.a.	0.19		1.1		
Leukopenia frequencies [90] (%) (WBC < 3500/mm^3^, Chinese)	15.1	n.a.	30.8	35.0	49.1	42.2	100	n.a.	100	n.a.	100	100
p.Arg139Cys test result	Normal (Arg/Arg)	Normal (Arg/Arg)			Medium (Arg/Cys)		Medium (Arg/Cys)			Low (Cys/Cys)		

NUDT15, Nucleoside diphosphate-linked moiety *X*-type motif 15; WBC, white blood cells; n.a., data not available

^a^ Diplotype frequencies in Korean were estimated using haplotype frequencies reported by Kim et al. [89]

^b^ Diplotype frequencies in Japanese were estimated using single nucleotide polymorphism frequencies from 3.5KJPN data by the Tohoku Medical Megabank Project (https://ijgvd.megabank.tohoku.ac.jp/) [51, 52, 91]

More recently, an additional three variants, namely, p.Arg34Thr, p.Lys35Glu, and p.Gly17_Val18del, were observed in five children with ALL in Singapore, Taiwan, and the USA [91]. All of the children experienced reduced tolerance to 6-MP, and p.G17_V18del was only observed in children of European or African ancestries. This deletion is the first functional variant reported in European and African populations. Three other rare functional variants, i.e., p.Met1Thr (lost of start codon), p.Arg10Trp, and Gly47Arg, were observed in a Japanese cohort [50, 51], but their enzyme activities and association with thiopurine-induced adverse events are unknown. Many rare variants and additional haplotypes may exist, such as TPMT.

## Prospects for clinical application of NUDT15 genotyping

All results to date indicate that thiopurine-induced leukopenia and severe alopecia are inevitable in patients homozygous for the p.Arg139Cys mutation. Consequently, p.Arg139Cys is a robust candidate for clinical applications aimed at predicting severe leukopenia and alopecia in patients with IBD. It remains unclear whether either the genotype of p.Arg139Cys or the diplotype of NUDT15 are preferable for clinical applications. If only p.Arg139Cys is tested, the activities of NUDT15 are expected to be normal (Arg/Arg), medium (Arg/Cys), or low (Cys/Cys) depending on the genotype. However, some diplotypes, such as *1*5,*1*6,*3*5, and *2*6, which have medium or low enzyme activities in vitro, cannot be correctly identified (Table 1). Chao et al. reported that the predictive sensitivity of NUDT15 p.Arg139Cys was 49.2% in their cohort of Chinese patients with IBD but that combined analysis with Val18Ile and p.Val18_Val19insGlyVal to determine diplotypes by detecting haplotypes *5 and *6 could increase the sensitivity to 55.4% [89]. This was the first study to examine the diplotype-based risk of thiopurines for IBD. However, the results should be interpreted with caution. Curiously, in the study of Chao et al. [89] the sensitivity of NUDT15 p.Arg139Cys for detecting leukopenia was lower than previously reported [72, 73, 82], possibly due to differences in leukopenia definitions in the various studies. In their study, Chao et al. [89] defined leukopenia as a white blood cell (WBC) count of < 3500/mm^3^, which is relatively higher than levels reported elsewhere. Higher grade leukopenia (WBC < 2000/mm^3^) is clinically important because of severe associated infectious complications and high mortality rates associated with IBD [92]. It is unclear which grade of leukopenia the patients with each diplotype had, and it is also important to consider if they had alopecia given its severity, prolonged recovery times, and associated cosmetic issues.

For clinical applications, it is important to clearly decide on the target of the pharmacogenetic tests. Additionally, the time, effort, and costs of genotyping are important factors to take into consideration. Given the diplotype frequencies in Chinese patients with IBD and the estimated frequencies, based on haplotype frequencies, in the Korean and Japanese cohorts, undetectable diplotypes using the test for p.Arg139Cys are infrequent, and especially rare (< 1%) among low activity diplotypes (*2*6, *2*5, and *3*5). Therefore, we need to evaluate the genotyping-associated costs to identify these patients and examine their detailed clinical information, such as adverse event types and grades. If the target of the pharmacogenetic test is to screen for patients who will have severe leukopenia and alopecia, detecting p.Arg139Cys may suffice. If the target is to determine initial doses of thiopurines, detecting the diplotype of patients might be helpful. However, there is insufficient evidence, and further analyses with large cohorts and detailed clinical data are needed to confirm the clinical effects of these variants.

## Conclusion

The mechanisms of thiopurine toxicity in Asian populations have been unclear for a long time, but were recently clarified by a valuable genome-wide association study in an Asian population. Given existing data pertaining to TPMT in a population of patients of European ancestry, testing the genotype or diplotype of NUDT15 is a good candidate in clinical applications for predicting thiopurine toxicity in Asian and Hispanic populations. However, further analyses will be needed to determine how and which variants should be genotyped, taking into account the allele frequencies in the target population. The costs and benefits of genotyping also require consideration. NUDT15 is a good model for showing that population-specific variants cause population-specific drug intolerance. These results suggest that further population-specific pharmacogenetic studies are indicated.
